# Reproducibility of Brain Morphometry from Short-Term Repeat Clinical MRI Examinations: A Retrospective Study

**DOI:** 10.1371/journal.pone.0146913

**Published:** 2016-01-26

**Authors:** Chung-Yi Yang, Hon-Man Liu, Shan-Kai Chen, Ya-Fang Chen, Chung-Wei Lee, Lee-Ren Yeh

**Affiliations:** 1 Department of Medical Imaging, E-Da Hospital, I-Shou University, Kaohsiung, Taiwan; 2 Department of Medical Imaging, National Taiwan University Hospital and National Taiwan University College of Medicine. Taipei, Taiwan; 3 Center for Dynamical Biomarkers and Translational Medicine, National Central University, Chungli, Taiwan; 4 Department of Medical Imaging and Radiological Sciences, I-Shou University, Kaohsiung, Taiwan; Rutgers University, UNITED STATES

## Abstract

**Purpose:**

To assess the inter session reproducibility of automatic segmented MRI-derived measures by FreeSurfer in a group of subjects with normal-appearing MR images.

**Materials and Methods:**

After retrospectively reviewing a brain MRI database from our institute consisting of 14,758 adults, those subjects who had repeat scans and had no history of neurodegenerative disorders were selected for morphometry analysis using FreeSurfer. A total of 34 subjects were grouped by MRI scanner model. After automatic segmentation using FreeSurfer, label-wise comparison (involving area, thickness, and volume) was performed on all segmented results. An intraclass correlation coefficient was used to estimate the agreement between sessions. Wilcoxon signed rank test was used to assess the population mean rank differences across sessions. Mean-difference analysis was used to evaluate the difference intervals across scanners. Absolute percent difference was used to estimate the reproducibility errors across the MRI models. Kruskal-Wallis test was used to determine the across-scanner effect.

**Results:**

The agreement in segmentation results for area, volume, and thickness measurements of all segmented anatomical labels was generally higher in Signa Excite and Verio models when compared with Sonata and TrioTim models. There were significant rank differences found across sessions in some labels of different measures. Smaller difference intervals in global volume measurements were noted on images acquired by Signa Excite and Verio models. For some brain regions, significant MRI model effects were observed on certain segmentation results.

**Conclusions:**

Short-term scan-rescan reliability of automatic brain MRI morphometry is feasible in the clinical setting. However, since repeatability of software performance is contingent on the reproducibility of the scanner performance, the scanner performance must be calibrated before conducting such studies or before using such software for retrospective reviewing.

## Introduction

Brain morphometry is an important assessment used in neuroscience research. Chronological changes in brain morphology may be related to aging, neurodegenerative disease, brain insults, or treatment effects [1–5]. Magnetic resonance imaging (MRI), a widely used non-invasive, clinical, diagnostic tool, can provide excellent brain tissue contrast. The high resolution of brain MRI also makes MRI-derived morphometric data ideal for clinical or research purposes. However, due to the complexity of brain morphology, accurate neuroanatomical measurement is difficult and time-consuming even for trained experts. For studies that require quantitative assessment of a large number of subjects or brain regions, automated methods play an important role in providing rapid and robust segmentation, while also minimizing human inter- and intra-rater variability.

Quantitative imaging biomarkers can serve as indicators of normal biological processes, pathogenic processes, or responses to therapeutic intervention [6, 7]. Among them, brain MRI morphometry is a promising imaging biomarker for early detection or diagnosis of neurodegenerative and psychiatric disorders [8–11]. Several software packages have been developed that provide automated segmentation and measurement of brain morphometry [12–15].

Prospective longitudinal studies of morphometric changes within the brain require both accuracy and reproducibility of automated morphometric measures across sessions. While the accuracy validation of automated neuroanatomical measures against regional manual measurements has been performed [13, 16], some studies have reported variable accuracy and reliability across sessions involving MRI-derived measures on the same subjects, and the sources of variance and bias have been investigated [17–24]. The reliability of brain morphometric measures across sessions can be influenced by subject-related, instrument-related, and image processing-related factors. Clinically, we usually need to retrospectively review previously acquired serial brain MRIs of patients to determine if any chronological change in brain morphometry has occurred. However, images are usually reported in a qualitative manner, so that there is little need for well-controlled image acquisition parameters other than image quality. It is also commonplace for hospitals to have multiple scanners and there is no clinical stipulation for follow-up scans to be performed on the same scanner, although such a practice is preferred. In consideration of the need for quantitative comparison of objective measures of disease progression or response to therapeutic treatment, it is important to evaluate the feasibility of using automated segmented measures from previously acquired clinical brain MRI scans for comparison purposes.

Our study had as its goal to validate the cross-session agreement of automatic segmented MRI-derived measures obtained using FreeSurfer software in a group of subjects with normal-appearing MR images. While this study was performed using FreeSurfer, the methodology could also be applied to other software packages and other images.

## Materials and Methods

### Ethics Statement

This study was approved by the National Taiwan University Hospital Institutional Review Board (IRB: 201207033RIC) and the requirement to obtain informed consent was waived due to its retrospective nature. The patient records were anonymized and de-identified prior to analysis.

### Subjects

From December 2011 to December 2013, 14,758 subjects (all older than 20 years of age) received brain MRI examinations in the Department of Medical Imaging at our hospital. These subjects included those referred from clinical departments (clinics, wards, and the emergency department) and volunteers for health screening. There were 2,299 subjects who had more than one brain MRI exam in this period. To limit the variance caused by instrument-related factors, only subjects who received repeat MRI studies on the same scanner were selected for this study.

A neuroradiologist (HML) with 30-years of experience carefully reviewed the medical history and images of all subjects. After excluding cases of dementia, psychiatric disorders, neoplastic disease, ischemic or hemorrhagic brain insults, infectious disease, prior surgical intervention, or poor image quality, 36 subjects remained who were reported as neuroradiologically normal on repeat brain MRI using the same scanner.

### MRI Scanners

Five MRI scanners were used at our institute during 2011 to 2013. A routine brain MRI examination contained a 3D T1 pulse sequence on every subject. The sequences used for morphometric analysis in each MRI scanner are listed below. Please note that, although these are the default parameters in the scanning protocols, the technicians sometimes made minor adjustments at the time of scanning for practical reasons.

- **1.5T Signa Excite (GE Healthcare, Milwaukee, Wis)**: 3D T1 Spoiled Gradient-echo (SPGR, TR = 8.916 ms, TE = 3 ms, flip angle = 12°, FOV = 250.0 x 250.0 mm, slice thickness = 1.2 mm, contiguous 1.2 mm sections, 512 x 512 matrix, NEX = 0.75, voxel size = 0.49 x 0.49 x 1.2 mm); installed in 2005.
- **1.5 T Signa HDx (GE Healthcare, Milwaukee, Wis)**: 3D T1 Spoiled Gradient-echo (FastSPGR IrP, 3D COR 3D FSPGR IrP, TR = 9.904 ms, TE = 3.836 ms, flip angle = 12°, FOV = 240.0 x 240.0 mm, slice thickness = 1.2 mm, contiguous 1.2 mm sections, 256 x 256 matrix, NEX = 1, voxel size = 0.94 x 0.94 x 1.2 mm); installed in 2008.
- **1.5 T Sonata (Siemens, Erlangen, Germany)**: 3D T1 Spoiled Gradient-echo (FLASH, TR = 8 ms, TE = 3.12 ms, flip angle = 12°, FOV = 200.0 x 200.0 mm, slice thickness = 0.9 mm, contiguous 0.9 mm sections, 256 x 256 matrix, NEX = 1, voxel size = 0.78 x 0.78 x 0.9 mm); installed in 2001.
- **3.0 T Trio Tim (Siemens, Erlangen, Germany)**: 3D T1 Spoiled Gradient-echo (TurboFLASH, TR = 1720 ms, TE = 2.58 ms, flip angle = 9°, FOV = 220.0 x 220.0 mm, slice thickness = 1.2 mm, contiguous 1.2 mm sections, 512 x 512 matrix, NEX = 1, voxel size = 0.43 x 0.43 x 1.2 mm); installed in 2003.
- **3.0 T Verio (Siemens, Erlangen, Germany)**: 3D T1 Spoiled Gradient-echo (TurboFLASH, TR = 1450 ms, TE = 2.47 ms, flip angle = 9°, FOV = 240.0 x 240.0 mm, slice thickness = 1.1 mm, contiguous 1.1 mm sections, 640 x 640 matrix, NEX = 1, voxel size = 0.38 x 0.38 x 1.1 mm); installed in 2009.

FreeSurfer (http://freesurfer.net, Athinoula A. Martinos Center for Biomedical Imaging, Harvard-MIT, Boston) is a freely available open source software suite for processing and analyzing human brain MR images [12, 13]. With an array of algorithms and tools provided by FreeSurfer, automated or semi-automated analysis and visualization of structural, connectional and functional brain imaging data can be performed using a wide variety of hardware and operating systems. Such data includes segmentation, registration, cortical surface reconstruction, quantification of segmented structures, longitudinal processing, fMRI analysis, and tractography. FreeSurfer allows visualization of different computed measures (such as thickness, area, or volume) over the brain surface after automatic parcellation.

The 64-bit Linux version of FreeSurfer 5.1.0 was used in this study. In brief, the FreeSurfer’s preprocessing and segmentation were executed with the command “*recon-all -all*”. “*recon-all*” is a batch script which involves more than 30 reconstruction routines and can be summarized as follows (The details can be found in https://surfer.nmr.mgh.harvard.edu/fswiki/recon-all and http://surfer.nmr.mgh.harvard.edu/fswiki/ReconAllTableStableV5.1):

1. Motion correction and averaging.
2. Intensity correction and normalization.
3. Skull stripping.
4. Gray matter/white matter segmentation
5. Parcellation, labeling, and statistics.

All stages of cortical reconstruction were performed in a fully automated manner. To ensure the reproducibility of the computed data, no manual intervention was performed. After successful automated processing by FreeSurfer, inspection of the snapshots from the final results was performed to ensure that no obvious non-brain parts had been incorrectly segmented as brain tissue. Images of each subject were automatically processed with the longitudinal stream [25] in FreeSurfer to create a reliable unbiased within-subject template space and image, which were used in further processing steps, such as skull stripping, Talairach transforms, atlas registration, spherical surface mapping and parcellations. By using the FreeSurfer longitudinal stream, it can significantly increase reliability and statistical power [25]. After that, tabulated data of the segmented results were collected using two FreeSurfer summarizing scripts (i.e., *aparcstats2table* and *asegstats2table*).

After automatic segmentation and quantification, several types of measurement can be used for analysis, including area, volume, std (standard deviation of volume), thickness, thicknessstd (standard deviation of thickness), and meancurv (mean curvature). The neuroanatomical labels in FreeSurfer could be categorized according to the different measurements acquired (i.e., area, thickness, or volume), the sidedness (left, right), cortical parcellation atlas (Desikan-Killiany Atlas [aparc] or Destrieux Atlas [a2009s]), and anatomical locations.

In this study, area, volume, and thickness measurements of segmented anatomical labels from the two MRI scans for each subject were selected for label-wise longitudinal analysis. Among them, the volumes of cortical gray matter (label: CoretexVol), deep gray matter (label: SubCortGrayVol), and white matter (label: CorticalWhiteMatterVol) were selected for analysis of the global measure of volume. For cortical area measurements, there were 150 labels for the Destrieux Atlas and 70 labels for the Desikan-Killiany Atlas. For cortical thickness measurements, there were 298 labels for the Destrieux Atlas and 138 labels for the Desikan-Killiany Atlas. For volume measurements, there were 148, 68, 76, and 55 labels for Destrieux Atlas, Desikan-Killiany Atlas, subcortical white matter, and miscellaneous structures, respectively. In total, there were 1003 labels for all parcellation/segmentation types of area, thickness, and volume measurements generated by FreeSurfer.

### Statistical Analysis

Intraclass correlation coefficient (ICC) [26–28] is a descriptive statistic commonly used to assess the reliability when quantitative measurements are organized into groups. It can be interpreted as the proportion of the total variance that is due to variation between groups. It measures the degree to which the units in the same group approximate each other. ICC will approach 1.0 when within-target variation is small, suggesting consistent results from each measurement. ICC will be negative when the between-target variation is relatively small compared to the within-target variation. An examination or a test is considered reliable if consistent results can be obtained under similar methodology. There are two kinds of reliability: consistency or absolute agreement. Because our targets of interest are absolute measurements (e.g. area, thickness, and volume), ICC was computed to assess the reliability of the results across sessions using a two-way random model with measures of absolute agreement. To approximate the actual distribution of ICC values, we employed 10,000 bootstrap-based resampling with an equal sample size (N = 20) for each model to computed means and 95% confidence intervals.

The Wilcoxon signed rank test is a non-parametric paired difference test. It is used to test the null hypothesis (i.e., the median of the differences between the paired observations is zero) versus the alternative hypothesis (i.e., the median is not zero). While the sample size was small, it can be used as an alternative to the paired t-test. After parcellation of the two sessions of brain MRI data, comparison of the paired segmented results was performed using the Wilcoxon signed rank test assuming a 5% and a 1% level of significance (α = 0.05 and 0.01, respectively).

Bland-Altman (BA) analysis is a graphical method used in analyzing the agreement between two measurement methods [29, 30]. This method plots the difference against the average of each data pair between two methods of measurement. The 95% limits of the mean difference were calculated and plotted for visual judgment of agreement. It was expected that 95% of differences were included in the 95% limits between the two measurement methods. A smaller range between these two limits indicates better agreement but the threshold of the range depends on the clinical context. In this study, Bland-Altman analysis was used for a global measure of selected brain volume.

A dimensionless measure of absolute percent change in segmentation results for a structure with respect to its average was used to estimate the variability error. The across-session variability error was estimated as follow:

$$\epsilon \;=\;\frac{\left|{\text{x}}_{1}-{\text{x}}_{2}\right|}{\left({\text{x}}_{1}+{\text{x}}_{2}\right)/2}\times 100\text{\%}$$

Kruskal-Wallis test, a non-parametric method used for comparing two or more samples that are independent, was used to test the MRI model effect on the ICC and variability error. All statistical analyses were performed using R version 3.1.2 [31].

## Results

### Subjects and MRI

After careful exclusion of any known brain disease or morphologic abnormality, 36 subjects remained who had repeat brain MRI scans using the same machine. The MRI results of Signa HDx were not analyzed in this study because only two subjects had repeat scans during this period. A total of 34 subjects (15 males and 19 females) representing 68 sessions of MRI scanning were included in this study. Their median age was 54.5 years (range: 34–85 years) (Table 1).

**Table 1:** Demographics of the 34 subjects.

Model	Subjects	Age (year)*	Gender**	MRI sessions	Follow up interval (month)*
**Signa Excite**	12	55.5 (42–75)	5/7	24	5.3 (2.8–12.6)
**Sonata**	5	52 (34–59)	3/2	10	5 (3.6–9.7)
**TrioTim**	6	61 (51–79)	2/4	12	6.4 (0.5–12.3)
**Verio**	11	54 (50–85)	9/2	22	12.4 (10.8–15.5)

* Median (range);

** Female/Male

### Intraclass Correlation Coefficient

**Fig 1:**
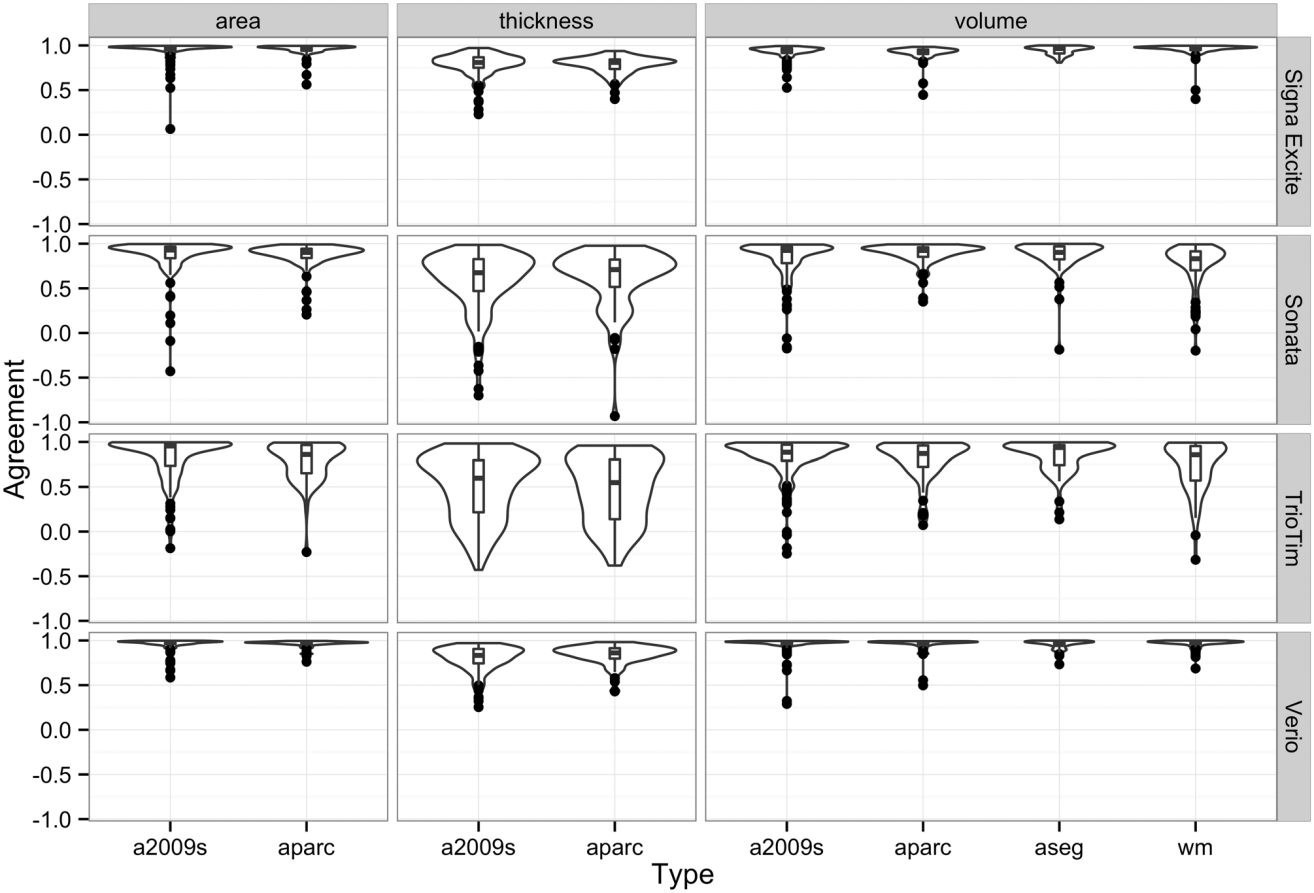
The violin plot of label-wise agreement (ICC) for each MRI scan. The bootstrapped ICCs of each label from each measurement produced by FreeSurfer are summarized in Fig 1. In addition to the information in the box plot, the shape of the distribution visualized in the violin plot helps to detect clusters or bumps within a distribution. This plot reflects the density of label-wise ICC among different scanners grouped by measurement and parcellation/segmentation types. The distribution of the density plot reveals the portion from different levels that are in agreement within each group. Abbreviations: a2009s, Destrieux Atlas; aparc, Desikan-Killiany Atlas; wm, subcortical white matter; aseg, miscellaneous structures.

The label-wise ICCs from the repeat MRI scans are shown in Fig 1. For each measurement, the ICCs in Signa Excite and Verio were generally in better agreement compared with the results from Sonata and TrioTim. Poor agreement was seen for the thickness and white matter volume measurements obtained using TrioTim. The mean and standard deviation of the ICC for each MRI model were 0.85 ± 0.16 (Signa Excite), 0.68 ± 0.34 (Sonata), 0.66 ± 0.33 (TrioTim), and 0.87 ± 0.17 (Verio). Kruskal-Wallis test revealed a significant MRI model effect on the ICCs (*p* < 0.001).

### Wilcoxon Signed Rank Test

**Fig 2:**
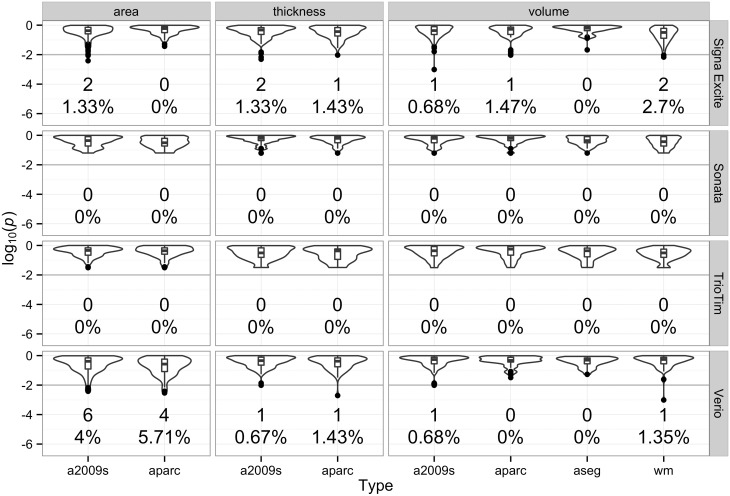
The violin plot of the *p* value of label-wise Wilcoxon signed rank test for each MRI scan. The *p* value is presented as log_10_(*p*). This plot reflects the distribution of label-wise *p* values between different machines by different measurement type and parcellation/segmentation type. The number and percentage of the regions of statistical significance (*p* < 0.01 or log_10_(*p*) < -2) were also labeled below the violin distribution. Abbreviations: a2009s, Destrieux Atlas; aparc, Desikan-Killiany Atlas; wm, subcortical white matter; aseg, miscellaneous structures.

**Fig 3:**
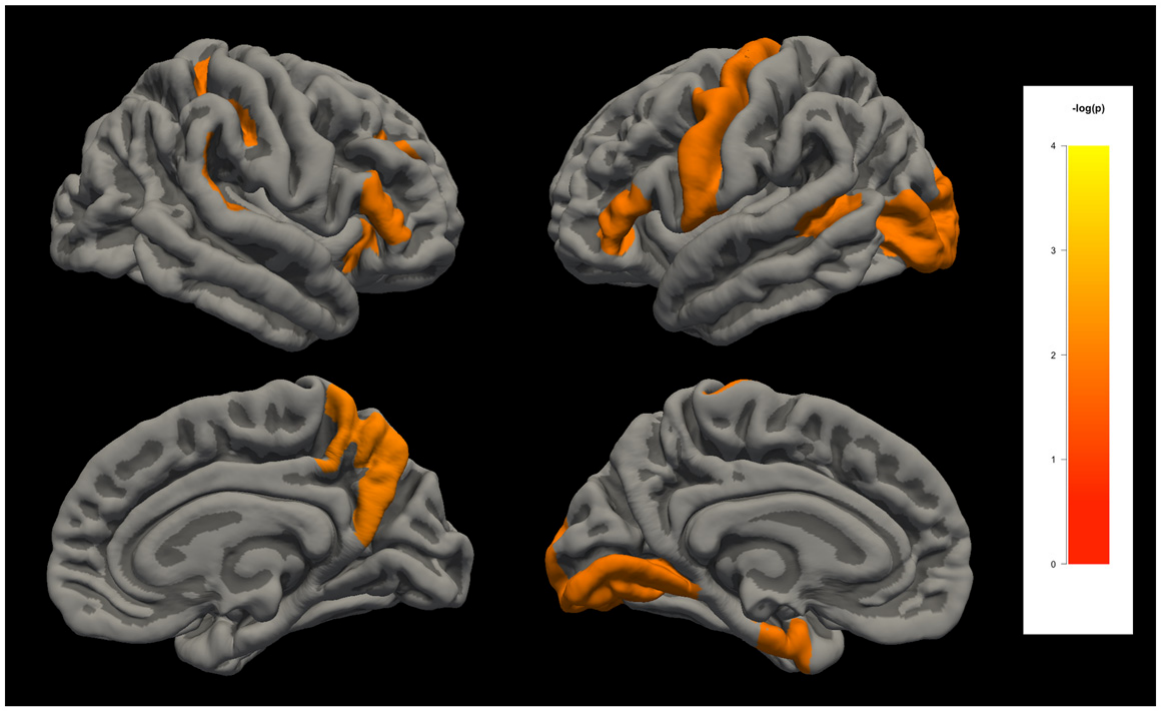
The regions of significant difference using Wilcoxon signed rank test are labeled and colored on the cortical surface for visualization.

The *p* values of the Wilcoxon signed rank test for each label of each measurement produced by the FreeSurfer are summarized in Fig 2. Among all labels, significant differences were found across repeat MRI scans in 121 (12.1%) and 23 (2.3%) labels for 5% and 1% significance level test, respectively. The labels with significant differences (α = 0.01) are summarized in Table 2 and Fig 3, especially some measurements at some measurements around the bilateral central gyri of the Verio model and scattered labels of the Signa Excite model.

**Table 2:** Regions showing significant differences.

model	ICC	p	Wilcoxon test (*p*)	measurement	hemisphere	label	type
**Signa Excite**	0.99	0.00	0.01	area	rh	G_front_inf-Triangul	a2009s
**Signa Excite**	0.95	0.00	0.00	area	rh	S_circular_insula_ant	a2009s
**Signa Excite**	0.82	0.01	0.01	thickness	rh	G_temp_sup-Plan_tempo	a2009s
**Signa Excite**	0.85	0.00	0.00	thickness	rh	S_front_middle	a2009s
**Signa Excite**	0.89	0.00	0.00	volume	rh	S_circular_insula_ant	a2009s
**Signa Excite**	0.84	0.00	0.01	thickness	lh	bankssts	aparc
**Signa Excite**	0.95	0.00	0.01	volume	lh	entorhinal	aparc
**Signa Excite**	0.93	0.00	0.01	volume	rh	wm-rh-parsorbitalis	wm
**Signa Excite**	0.97	0.00	0.01	volume	rh	wm-rh-superiortemporal	wm
**Verio**	0.97	0.00	0.00	area	lh	G_front_inf-Triangul	a2009s
**Verio**	0.96	0.00	0.00	area	lh	G_oc-temp_med-Lingual	a2009s
**Verio**	1.00	0.00	0.00	area	lh	S_occipital_ant	a2009s
**Verio**	0.99	0.00	0.01	area	rh	G_precuneus	a2009s
**Verio**	0.99	0.00	0.01	area	rh	S_circular_insula_ant	a2009s
**Verio**	0.98	0.00	0.00	area	rh	S_postcentral	a2009s
**Verio**	0.76	0.01	0.01	thickness	lh	G_precentral	a2009s
**Verio**	0.97	0.00	0.01	volume	lh	G_precentral	a2009s
**Verio**	0.98	0.00	0.01	area	lh	lateraloccipital	aparc
**Verio**	0.97	0.00	0.00	area	lh	lingual	aparc
**Verio**	0.99	0.00	0.00	area	lh	parstriangularis	aparc
**Verio**	0.99	0.00	0.01	area	rh	precuneus	aparc
**Verio**	0.88	0.01	0.00	thickness	lh	precentral	aparc
**Verio**	0.96	0.00	0.00	volume	lh	wm-lh-parstriangularis	wm

### Bland-Altman Analysis (Global Measure of Volume)

**Fig 4:**
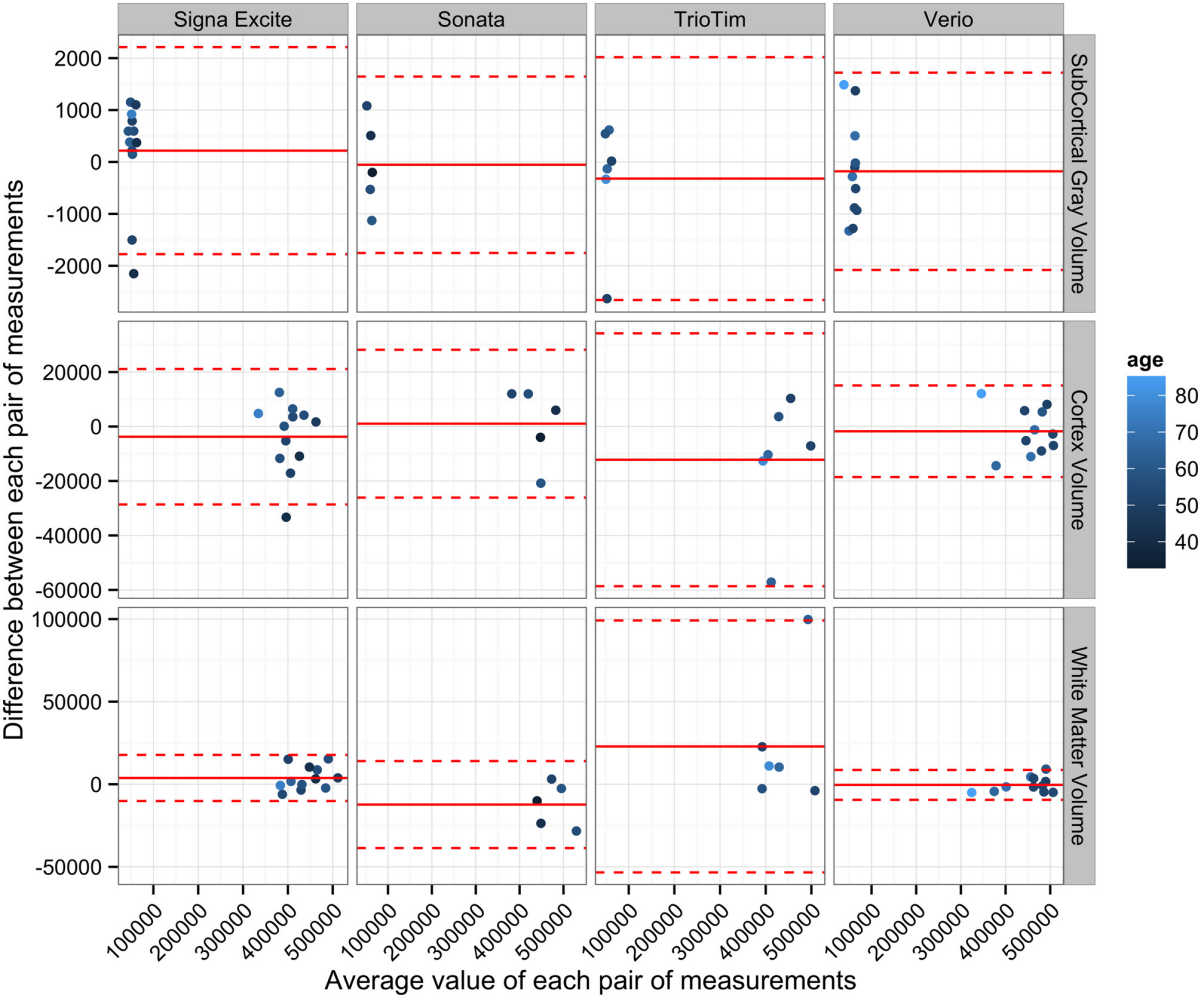
Results of the Bland-Altman analysis comparing results obtained from repeat MRI scans using different MRI models and selected global measures of brain volume. These plots represent the difference between repeat MRI scans against the average value for each pair of measurements. A continuous line and two dotted lines represent the mean difference between each pair of measurements and the limits of agreement, respectively, for each BA plot.

In addition to the ICC and Wilcoxon signed rank test, selected global measures of brain volume (cortical gray matter, subcortical gray matter, and white matter) were also analyzed using the Bland-Altman (mean-difference) plot of each pair of measurements (Fig 4 and Table 3). Greater mean differences and wider agreement intervals were observed in the TrioTim model.

**Table 3:** Bland-Altman analysis of global measures of volume obtained from repeat MRI scans.

Measurement	Model	Mean differences (mm^3^)	Standard deviation of differences (mm^3^)	Standard error of mean (mm^3^)	N
**SubCortical Gray Volume**	Signa Excite	218.0	1017.4	1513	12
**SubCortical Gray Volume**	Sonata	-54.0	866.9	2184	5
**SubCortical Gray Volume**	TrioTim	-320.5	1193.3	2046	6
**SubCortical Gray Volume**	Verio	-180.1	969.4	2524	11
**Cortex Volume**	Signa Excite	-3754.7	12690.8	9201	12
**Cortex Volume**	Sonata	1038.5	13845.9	16745	5
**Cortex Volume**	TrioTim	-12213.9	23688.8	15838	6
**Cortex Volume**	Verio	-1748.3	8576.9	15438	11
**White Matter Volume**	Signa Excite	3802.5	7123.4	12171	12
**White Matter Volume**	Sonata	-12303.8	13437.7	16267	5
**White Matter Volume**	TrioTim	22878.6	38911.4	20862	6
**White Matter Volume**	Verio	-384.1	4614.4	17312	11

### Across-Session Variability

The across-session variability errors of all segmentation results were computed. Among them, the results of three global measures of brain volume (cortical gray matter, subcortical gray matter, and white matter) are summarized in Table 4. In each MRI scanner, the mean reproducibility error was estimated as a mean across all subjects. The last column shows the effects of analysis on the various structures by averaging the across-MRI model reproducibility errors across MRI models. Although no significant MRI model effect was found among these selected global measures, significant MRI model effect was shown on 125 (12.4%) and 14 (1.4%) labels among all segmentation results for 5% and 1% significance level tests, respectively.

**Table 4:** Volumetric reproducibility error for the various MRI models from FreeSurfer segmentation. Each cell represents the mean reproducibility errors (percent absolute difference relative to the mean) across subjects within each MRI scanner. Among these three selected global volumetric measures, there was no significant MRI model effect observed (Kruskal-Wallis test). Although not significant, large percent absolute differences were seen within the TrioTim model (cortex and white matter volume), suggesting the existence of outliers.

Volume	Signa Excite	Sonata	TrioTim	Verio	Mean error across MRI models (%)	p*
SubCortical Gray Volume	1.55 ± 1.06	1.18 ± 0.73	1.36 ± 1.85	1.45 ± 1.18	1.43 ± 1.18	0.77
Cortex Volume	2.34 ± 2.29	2.55 ± 1.52	4.03 ± 4.89	1.73 ± 1.12	2.47 ± 2.58	0.69
White Matter Volume	1.34 ± 1.21	2.82 ± 2.38	5.43 ± 7.49	0.87 ± 0.53	2.13 ± 3.55	0.12

* Kruskal-Wallis test across MRI models

## Discussion

This study evaluated the automatic segmented results obtained using FreeSurfer on a group of normal subjects who received repeat MRI scans over a short time interval (up to 15.5 months). Unlike most prospective studies, in which careful tuning of experimental conditions is expected, the subjects in this study were retrospectively selected from a clinical database. Variable degrees of reproducibility were noted using different MRI machines, shown as a wide range of ICCs (Fig 1). Although this is a study of short-term repeat MRI scans in normal subjects, significant differences were still found in some labels of different measures. Different variances were found in the results of the BA analysis from different machines.

Comparison of longitudinal follow-up results from different MRI systems is possible, however, there are some limitations regarding image quality, which may be influenced by acquisition sequences, magnetic field strength, scanner software, or the type of scanner used [18, 22]. Although it has been suggested that using registration-based algorithms can provide better reproducibility, for longitudinal follow-up, MRI acquisitions should be performed at the same imaging site [18]. The automatic segmentation software used in this study, FreeSurfer, is implemented with segmentation-based algorithms. In this study, repeat longitudinal follow-up examinations in a group of neuroradiologically normal subjects were performed on the same MRI scanner, however, less agreement and large mean differences in the morphometric data across sessions were found with certain MRI scanners, as shown in Figs 1 and 4. While the vast majority of ICCs for each label from repeat scans were high in scans from the Signa Excite and Verio MRI scanners, a certain portion of low ICCs were noted in the Sonata and TrioTim models. Varying degrees of agreement between scanner types were also found in this study using Bland-Atman analysis and, similarly, a MRI model effect on the absolute percent difference was revealed in segmentation results using the Kruskal-Wallis test. Individual comparison with normal databases is important for the purposes of reporting, as the confidence interval of normal data can serve as a reference to improve the accuracy of interpretation. While many different scanners could be used to create an MRI database, considerable across-scanner variability might exist and, thus, it is necessary to quantify the differences and to determine the significance of the variability using the reference data.

Although both Signa Excite (GE) and Sonata (Siemens) are 1.5T MRI models, the agreement and variance in generated FreeSurfer results were quite different between models. Better agreement and smaller variance could be seen in the scans from the Signa Excite system. A difference in scanning parameters or vendors, and magnetic field inhomogeneity due to aging of the machine may have contributed to these differences. On the other hand, although TrioTim and Verio are both 3T MRI models from the same manufacturer (Siemens), the ability to reproduce quantifiable results from the two machines differed significantly.

While there are various accelerating methods used for high-quality high-resolution 3D T1 images, MP-RAGE and IR-SPGR are the sequences of choice for brain morphometry because of their superior signal to noise ratio and gray-white matter contrast. The selection of acquisition sequences for regular MRI scanning in a clinical site relies on a balance between scanning time, tissue contrast, resolution, sensitivity to motion, and availability of the sequence. For a clinical examination, the sequences are optimized to detect abnormalities rather than to obtain the best segmentation results by automated processing software. It is common not to use MP-RAGE or IR-SPGR for regular brain MRI scanning. Although the sequences used in this study (SPGR, FLASH, TurboFLASH) are alternatives to MP-RAGE/IR-SPGR [32], the gray-white matter contrast may effect the ability of the segmentation software to optimally perform. Thus, it is crucial to choose an optimized sequence when using brain morphometry metrics as a quantitative imaging biomarker in clinical trials, or the suboptimal segmentation may influence the accuracy of the results.

Results of neuroanatomical labeling using two cortical parcellation systems, Desikan-Killiany Atlas [33] and Destrieux Atlas [34], were provided in FreeSurfer. Each cortical vertex of the brain was classified using gyral-based mapping for the Desikan-Killiany Atlas, which contains 68 gyral-based labels, 34 for each hemisphere. For the Destrieux Atlas, each vertex can be categorized as sulcal or gyral, and then subparcellated into 148 labels, 74 for each hemisphere. Since there were more labels and a more complicated classification system for use with the Destrieux Atlas, it was more difficult to maintain accuracy and consistency in segmentation results. Compared with the Desikan-Killiany Atlas, the increased number of labels used by the Destrieux Atlas had a significant effect, based on the Wilcoxon signed rank test.

The MRI radiographers are trained to acquire high quality scans for lesion detection in their daily practice. However, it is possible that a more diligent MRI technician who ensures higher quality scans on one particular scanner while others might accept minor quality degradation as long as the quality is good enough for a radiologist to make a diagnosis. After reviewing the original MR images of the outliers shown in Fig 4, a small amount of blurring caused by motion artifact was found in the image of one outlier, and this can be avoided by a sophisticated radiographer in a prospective quantitative brain morphometric study. Given the retrospective nature of this clinical study, between-scanner differences could also be partly contributed by inter-radiographer variability.

The ultimate goal of medical researchers is to apply the observed findings to the clinical situation, and such an application relies on accuracy and reproducibility of the segmented results. Appreciable differences in subcortical brain volumes in within-a-day repeat MRI scans on the same subject could still be found, even when using the same scanner and acquisition parameters, because of changes in image orientation, pre-scan parameters, and magnetic field instability [19]. Similar scan-rescan measurement differences and variability in scanning parameters/environments have been reported [22, 35]. In the clinical setting, more variability will be encountered and, therefore, a preliminary validation of data accuracy and reproducibility is necessary. Otherwise, false positive labels of significant differences will be reported if an unreliable MRI system is used. With the implementation of automated segmentation methods, we can quantify and determine the significance of the effect caused by scanner-related factors. Furthermore, we can use the same processing pipeline to investigate whether these fundamental differences can be removed or minimized. An estimated scanner-related effect should be used for the correction of segmentation results.

Our study had several limitations including its retrospective nature. In addition, this study was conducted at a single institution. Future prospective studies performed at multiple institutions involving larger cohorts are necessary to confirm our results. The inclusion of “normal” subjects relied on our neuroradiologist’s judgment regarding the absence of pathological findings on the MR images, however, it is possible that subjects with neurodegenerative disease did not exhibit pathological findings but, rather, morphometric changes within the brain. Therefore, a prospective validation study on normal subjects should be conducted. Theoretically, segmentation of isometric voxels should be less sensitive to changes in image orientation compared with non-isometric voxels. Non-isometric or near-isometric voxel sizes were used in this study and the impact of voxel isometry on the accuracy and reproducibility of the segmentation should be further explored. In this study, the effect of MRI scanners was determined by detecting differences in each individual subject but certain regions of statistical significance in a particular individual may not be clinically relevant. Different pathologic conditions, such as neurodegenerative diseases, may have variable effects on brain morphology and the size of the effect should be determined by group analysis [36–38]. Furthermore, since discrepancies in the results derived from different segmentation methods have been shown to reach the same order of magnitude as volume changes in disease [18, 39], the effect of different methods on the reliability of the clinical MRI images should also be investigated in a future study.

## Conclusion

Short-term scan-rescan reproducibility of automatic brain MRI morphometry is feasible in the clinical setting, but validation of scan-rescan reproducibility for each MRI scanner is suggested before conducting such a study, or prior to using such software for retrospective reviewing.

## Supporting Information

S1 File The compressed archive containing the demographic data and segmentation results of thickness, area, and volume measurements by FreeSurfer. (ZIP)
